# Exploratory Study on Efficacy and Safety of Minocycline-Based Dual Therapy for *Helicobacter pylori* Eradication

**DOI:** 10.3390/pathogens14111121

**Published:** 2025-11-03

**Authors:** Wen Gao, Jingwen Li, Yuling Tian, Chaoyi Ge, Chi Wang, Jianxiang Liu, Yixuan Li, Hong Cheng

**Affiliations:** 1 GI Department, Peking University First Hospital, Beijing 100034, China; 2 School of Medicine, Tsinghua University, Beijing 100084, China; 3 F. Widjaja Inflammatory Bowel Disease Institute, Cedars-Sinai Medical Center, Los Angeles, CA 90048, USA

**Keywords:** *Helicobacter pylori*, vonoprazan, minocycline, amoxicillin, dual therapy, treatment

## Abstract

Background & Aims: This study aimed to evaluate the feasibility of vonoprazan–minocycline (VM) dual therapy for *Helicobacter pylori* infection. Methods: In this open-label RCT, 120 *H. pylori*-infected patients (60 treatment-naïve and 60 treatment-failure cases) were randomized to receive vonoprazan–amoxicillin dual therapy (VA: vonoprazan 20 mg b.i.d. + amoxicillin 1.0 g t.i.d., control group) or VM dual therapy (vonoprazan 20 mg b.i.d. + minocycline 100 mg b.i.d., test group) for 14 days. The primary outcome was eradication rates. Secondary outcomes included adverse effects (AEs). Results: As first-line treatment, eradication rates were 96.7% (VA) vs. 90.0% (VM) in intention-to-treat (ITT) analysis (*p* = 0.30) and 96.7% (VA) vs. 96.4% (VM) in per-protocol (PP) analysis (*p* = 0.96). For rescue treatment, eradication rates were 86.7% (VA) vs. 76.7% (VM) in ITT (*p* = 0.32) and 89.7% (VA) vs. 79.3% (VM) in PP (*p* = 0.28). Overall eradication rates were 91.7% (VA) vs. 83.3% (VM) in ITT (*p* = 0.17) and 93.2% (VA) vs. 87.7% (VM) in PP (*p* = 0.31). VM had a higher incidence of AEs than VA (30.0% vs. 10.0%, *p* = 0.006), with dizziness being the most common (18.3%). Conclusions: VM dual therapy was shown to be an effective and safe treatment option, demonstrating comparable eradication rates to VA dual therapy. While VM had a slightly higher incidence of AEs, they were generally mild and manageable. VM remained a valuable alternative for patients with penicillin allergies or amoxicillin resistance.

## 1. Introduction

*Helicobacter pylori* (*H. pylori*) eradication treatment is essential for decreasing the risk of gastritis, peptic ulcer, MALT lymphoma, and gastric cancer [1]. The introduction of potassium-competitive acid blocker drugs (P-CABs) has significantly transformed traditional *H. pylori* treatment, and they are recommended for use “in place of PPIs in eradication regimens for most patients with *H. pylori* infection” in the AGA clinical practice update in 2024 [2]. A recent network meta-analysis, which compared multiple randomized controlled trials (RCTs) on P-CAB-based dual, triple, and quadruple regimens with PPI-based dual, triple, and quadruple regimens, concluded that among the included regimens, the P-CAB-based dual therapy demonstrated good eradication rates, the best therapeutic efficacy, and a low incidence of adverse effects [3].

When we refer to P-CAB dual therapy, it usually refers to a regimen consisting of P-CAB and high-dose amoxicillin (1.0 g t.i.d., 3.0 g/day). Recent RCT studies have shown that this regimen can achieve eradication rates of 90% or higher, particularly in the Asia region [3]. Besides amoxicillin, tetracycline was one of the most effective antibiotics against *H. pylori*, with resistance generally less than 1.2~3.3% [4]. Our previous study on vonoprazan–tetracycline (VT) dual therapy was developed by modifying the classical bismuth-containing quadruple regimen, in which bismuth and metronidazole were omitted and the acid inhibitor was replaced with vonoprazan while maintaining the same tetracycline dosage. This simplified regimen achieved satisfactory eradication efficacy (94.5% in mITT, 95% CI 89.1–97.4%) in penicillin-allergic patients [5]. Tetracycline is not easily accessible in many countries and regions. Minocycline, a tetracycline derivative, was hypothesized to yield similar results when combined with vonoprazan (VM dual therapy). Moreover, several studies have demonstrated that substituting tetracycline with minocycline in classical quadruple therapy yields comparable results [6,7,8,9]. Based on these findings, the current vonoprazan–minocycline (VM) dual regimen was designed: removing bismuth and metronidazole and administering minocycline 100 mg twice daily- to evaluate whether similar or improved efficacy could be achieved with a simpler, well-tolerated combination [4]. We designed this small-scale exploratory study to evaluate the feasibility of VM dual therapy and provide a foundation for further research.

This study aimed to evaluate the efficacy and safety of 14-day vonoprazan and minocycline dual therapy (VM dual therapy) compared with vonoprazan and amoxicillin (VA dual therapy) as first-line and rescue treatment prospectively.

## 2. Materials and Methods

### 2.1. Study Design and Participants

This was a prospective, single-center, open-label, randomized controlled trial performed between June 2024 and May 2025 at Peking University First Hospital in China. The Ethics Committee of Peking University First Hospital approved this study (ethics approval number 2024Y207-002). All patients signed written informed consent. The reporting followed the CONSORT guidelines for randomized controlled trials. This trial was registered on chictr.org.cn (ChiCTR2400086342). The study data were accessible to the co-authors, who reviewed and gave their approval for the final manuscript.

Eligible patients were those aged between 18 and 80 years, with *H. pylori* positive (naïve in treatment or had failed in previous treatment). Exclusion criteria: Patients with a history of *H. pylori* eradication using a VA dual therapy or a regimen containing minocycline; Use of antibiotics, bismuth agents, or acid suppressants within 4 weeks before treatment; Pregnant or lactating women; Patients with other severe concurrent diseases that may interfere with the study evaluation, such as severe liver disease, heart disease, kidney disease, malignancy, or alcoholism; Hypersensitivity to any of the study drugs (vonoprazan, amoxicillin, minocycline) or their components; Participation in other drug studies within 3 months prior to the use of the investigational drugs; Patients unable to properly communicate their symptoms, such as those with psychiatric disorders or severe neurosis, or those unable to cooperate with the study; Other conditions deemed by the investigator to make the patient unsuitable for participation in this study.

After enrollment, all participants had their *H. pylori* infection confirmed through a positive ^13^C-urea breath test. (UBT, 75 mg ^13^C-urea, Shenzhen Zhonghe Headway Bio-Sci & Tech Co., Ltd., Shenzhen, China) [10].

### 2.2. Randomization and Interventions

The dosage regimen of vonoprazan (20 mg twice daily) and minocycline (100 mg twice daily) was determined according to previous clinical experience with minocycline-based quadruple therapy and available pharmacodynamic evidence indicating that these doses maintain sufficient antimicrobial concentrations while minimizing adverse reactions [4,8,9,10].

After enrollment, patients were randomly assigned to receive either open-label VM dual therapy (test group), consisting of vonoprazan 20 mg twice daily (20 mg/tablet, Takeda Pharmaceutical Co., Ltd., Tokyo, Japan) and minocycline 100 mg twice daily (50 mg/capsule, Hanhui Pharmaceutical Co., Ltd., Shanghai, China), or VA dual therapy (control group), consisting of vonoprazan 20 mg twice daily and amoxicillin 1000 mg three times daily (500 mg/capsule, the United Laboratories International Holdings Limited, Hong Kong, China), each for 14 days. Patients were asked to take vonoprazan 30 min before meals, while minocycline and amoxicillin be taken immediately after meals.

A computer-generated sequence with blocks of four was used for randomization. The sequence was enclosed in a sealed envelope and stored by an independent research assistant until the treatment assignments were determined. This was an open-label study, meaning both the physicians and patients knew the treatment allocations. However, the technician administering the ^13^C-UBT was blinded to the treatment assignments. Patients received detailed information regarding the medication regimen, potential side effects, and how to report adverse events. Patients were closely monitored for any signs of discomfort during and after the treatment period. To enhance adherence, educational support was offered during consultations, along with follow-up through both online and offline methods.

### 2.3. Trial Assessments

Positive judgment: Those with a positive ^13^C-urea breath test (UBT) within one month before enrollment. During the screening visit, demographic information and pertinent medical history were collected. Additionally, treatment-emergent adverse events (TEAEs) and any concomitant medications were recorded. TEAEs were documented for all patients who received at least one dose of the study drug. The primary endpoint was the eradication rate, determined by *H. pylori* status through the ^13^C-UBT at least 6 weeks after completion of treatment. Secondary endpoints included the frequency and severity of adverse events, along with adherence to the medication regimen.

### 2.4. Sample Size Calculation and Statistical Analysis

As a preliminary exploratory study, a small sample size was typically used to assess the feasibility, safety, and initial efficacy of a new treatment regimen. This approach helped to establish foundational data and experience for future larger-scale studies. Therefore, we designed two subgroups (first-line treatment group and rescue treatment group), with each subgroup divided into two groups, each consisting of 30 cases, resulting in a total sample size of 120 patients for this trial. This design allows for a comprehensive evaluation of the treatment effects within different populations while maintaining a manageable sample size for an exploratory study. The sample size ratio for the VM dual group versus the VA dual group was established at 1:1, utilizing a one-sided test with an α error of 0.025, a power of 80% (corresponding to a β error of 0.20).

Treatment effectiveness was evaluated in two patient groups: (1) the Intention-to-treat (ITT) analysis, which included all patients who completed at least 6 weeks of follow-up, considering those lost to follow-up as treatment failures; (2) the Per-protocol (PP) analysis, which focused on patients who adhered to at least 80% of the prescribed medication and completed the follow-up. Good adherence was defined as taking more than 80% of the prescribed medication.

Statistical analysis was performed by SPSS software (version 26), with a two-tailed *p*-value considered statistically significant if *p* < 0.05. Continuous variables are expressed as means ± standard deviation (SD), while categorical variables are presented as counts and percentages (%). Student’s *t*-test was applied to continuous variables, and the Chi-square test or Fisher’s exact test was used for categorical variables, depending on the situation.

## 3. Results

### 3.1. Patients Enrolled and Baseline Characteristics

**Figure 1:**
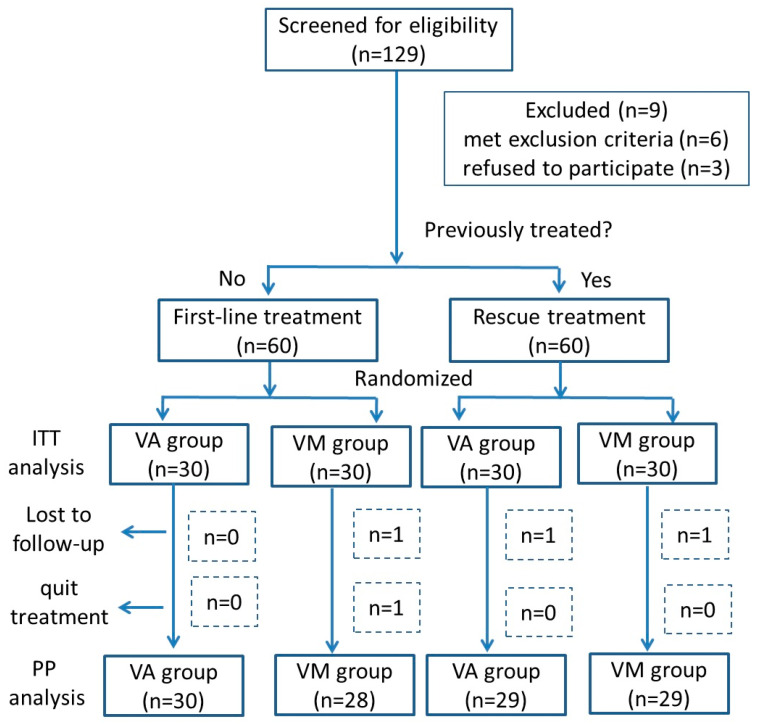
Flowchart of screening and recruitment of study subjects.

The enrollment process took place from June 2024 to May 2025 at Peking University First Hospital. Among the 129 patients screened for eligibility, 120 were randomized into the study, with 60 assigned to the VA dual therapy group and 60 to the VM group (Figure 1). The final follow-up was completed in May 2025.

The demographic and clinical characteristics of the study population are summarized in Table 1. In the VA dual therapy group, one patient was lost to follow-up and did not undergo the ^13^C-UBT, leading to classification as treatment failure in the ITT analysis and exclusion from the PP analysis. In the VM dual therapy group, three patients discontinued treatment (taking less than 80% of the prescribed tablets), but only one completed the ^13^C-UBT follow-up. The other two patients refused to complete the follow-up and were classified as lost to follow-up. Overall, four patients (one from the VA group and three from the VM group) were excluded from the PP analysis (Figure 1).

Cigarette smoking was defined by consumed >5 cigarettes a day or >1 cigarette pack/week consumed in the past 6 months.

Alcohol drinking was defined by >50 g of alcohol/day consumed in the past 6 months.

The family history of gastric cancer was considered positive if a first-degree relative (such as a parent, sibling, or child) had been diagnosed with gastric cancer, which is linked to a two- to three-fold increased risk of developing the disease [11].

### 3.2. Eradication of H. pylori Infection

The eradication rates are summarized in Table 2. For first-line treatment, the eradication rates for the VA and VM groups were 96.7% (29/30, 95% CI: 83.3–99.4%) and 90.0% (27/30, 95% CI: 74.4–96.6%) in the intention-to-treat (ITT) analysis, respectively (*p* = 0.30), 96.7% (29/30, 95% CI: 83.3–99.4%) for the VA group and 96.4% (27/28, 95% CI: 82.3–99.4%) for the VM group (*p* = 0.96) in the per-protocol (PP) analysis. For rescue treatment, the eradication rates were 86.7% (26/30, 95% CI: 70.3–94.7%) for the VA group and 76.7% (23/30, 95% CI: 59.1–88.2%) for the VM group in the ITT analysis (*p* = 0.32), 89.7% (26/29, 95% CI: 73.6–96.4%) for the VA group and 79.3% (23/29, 95% CI: 61.6–90.2%) for the VM group (*p* = 0.28) in the PP analysis. Overall, the eradication rates from the ITT analysis were 91.7% (55/60, 95% CI: 81.9–96.4%) for the VA group and 83.3% (50/60, 95% CI: 72.0–90.7%) for the VM group (*p* = 0.17). In the PP analysis, the eradication rates were 93.2% (55/59, 95% CI: 83.8–97.3%) for the VA group and 87.7% (50/57, 95% CI: 76.8–93.9%) for the VM group (*p* = 0.31). There was no significant difference in overall eradication rates between the two groups.

A total of 14 patients (4 in the VA group and 10 in the VM group) experienced treatment failure. The basic information and eradication status of these patients are summarized in Table 3. In the VA group, 4 patients experienced treatment failure, all of whom completed the full 14-day course of therapy. One patient switched to VM dual therapy after treatment failure and achieved eradication success. In the VM group, 10 patients experienced treatment failure. One of these patients discontinued treatment due to adverse effect and later switched to VA dual therapy, achieving successful eradication.

### 3.3. Adverse Events and Adherence

The adverse event analysis included 120 patients, of whom three refused re-evaluation via ^13^C-UBT and subsequent consultation, thus being recorded as lost to follow-up. Treatment-emergent adverse events (TEAEs) were reported in 30.0% (18/60) in the VM group compared to 10.0% (6/60) in the VA dual therapy group (*p* = 0.006) (Table 4).

The most common adverse event (AE) was dizziness, reported exclusively in the VM group (18.3%, 11/60; 2 males and 9 females) (Table 4). Most episodes of dizziness (9 cases) were mild and transient, resolving spontaneously within a few days or shortly after treatment cessation, without additional symptoms, while two cases of more severe dizziness accompanied by other discomforts discontinued due to AEs. In contrast, no cases of dizziness were observed in the VA group.

Three AEs in the VM group led to treatment discontinuation (5.0%, 3/60). In the VM first-line treatment group, a 45-year-old female developed dizziness, nausea, bradycardia, and hypotension by the third day. Her symptoms resolved two days after discontinuation with supportive intravenous fluids. Another case involved a 38-year-old female who experienced persistent dizziness, nausea, and vomiting from the first day of treatment, followed by bilateral breast tenderness on the fourth day. Symptoms resolved completely within two days after stopping the medication. In the VM rescue treatment group, a 28-year-old female developed a low-grade fever on the eighth day of treatment, which subsided after ibuprofen, but a skin rash appeared on the eleventh day, necessitating discontinuation. No recurrence of symptoms was observed after stopping the medication.

## 4. Discussion

Our previous study represented the first report on the VT dual therapy regimen, which combined vonoprazan (20 mg twice daily) and tetracycline (500 mg three times daily) administered over 14 days. This regimen had demonstrated both efficacy and safety as first-line treatment for *H. pylori* infection, particularly in populations with penicillin allergy [5,12]. This regimen offered advantages of a simple formulation with high eradication rates, fewer adverse events, and good adherence. However, the limited availability of tetracycline in many countries and regions, including China, has restricted its broader application.

Minocycline, a second-generation semi-synthetic tetracycline derivative introduced in 1967, shares similar pharmacological properties with tetracycline [13,14,15]. It had easy accessibility in most countries, promoting its widespread use, with twice-daily dosing regimen, which enhances patient adherence due to its extended half-life. It also had minimal food-drug interactions, ensuring consistent therapeutic outcomes [6]. Minocycline had been used to treat *H. pylori* infection since 2002, and numerous RCTs had evaluated the efficacy and safety of minocycline-based quadruple regimens [4,6,16]. These regimens had demonstrated good efficacy with acceptable side effects [8]. Resistance to minocycline was rare, with primary resistance reported at 0.7% (2/288), compared to 1.4% (4/288) for tetracycline [8,16], and secondary resistance was infrequent [15,17].

Minocycline-containing regimens were typically formulated as quadruple therapies, incorporating a gastric acid inhibitor, bismuth, minocycline, and an additional antibiotic. These regimens had proven effective in both first-line and rescue treatments [6,17]. These regimens achieved an ITT eradication rate of 83.6% (95% CI: 80.6–86.7%) as first-line treatment, 82.3% (95% CI: 79.5–85.2%) as rescue treatment, and 82.3% (95% CI: 79.7–85.1%) overall [6]. Among the combinations, nitroimidazole was the most frequently used antibiotic, then amoxicillin, while combinations with other antibiotics were less common [9,18].

Vonoprazan was chosen as the acid-suppressive agent in this study owing to its strong and stable ability to inhibit gastric acid secretion. In contrast to traditional proton pump inhibitors (PPIs), which require acid activation and show variability due to CYP2C19 genetic differences, vonoprazan acts directly and reversibly on the potassium-binding site of the gastric H^+^/K^+^-ATPase. This mechanism enables it to produce a rapid and sustained increase in intragastric pH. Pharmacodynamic investigations have demonstrated that vonoprazan can raise and maintain intragastric pH above 4.0 within a few hours after administration, thereby creating an optimal environment for antibiotic stability and *H. pylori* eradication [19]. Considering these pharmacological properties, together with our previous positive outcomes from vonoprazan–amoxicillin and vonoprazan–tetracycline dual therapies, vonoprazan was deemed appropriate for combination with minocycline in the current regimen.

In our study, the VA dual therapy was chosen as the control group due to its extensive validation in numerous RCTs, confirming its efficacy both as first-line and rescue treatment. The VM dual therapy in this study was designed to be served as a complementary alternative to the VA regimen. In this study, eradication rates in the VM group were lower than those in the vonoprazan–amoxicillin (VA) group both in first-line and rescue treatment. For first-line treatment, ITT analysis showed eradication rates of 90.0% in the VM group and 96.7% in the VA group (*p* = 0.30). For rescue treatment, eradication rates were 76.7% for the VM group and 86.7% for the VA group (*p* = 0.32). Overall, the eradication rates were 83.3% in VM group and 91.7% in VA group (*p* = 0.17). The VM therapy showed good efficacy as first-line treatment, while its eradication rate decreased in rescue treatment. Although this study found that the eradication rate of VM was slightly lower than that of VA, it still remained within an acceptable range. Future large-scale studies will help clarify whether this difference is statistically significant.

As presented in Table 3, the majority of eradication failures were observed among rescue patients. This finding aligns with the previous literature and our earlier observations. Patients undergoing rescue therapy have typically experienced prior eradication failure, most often attributable to antibiotic resistance of *H. pylori*. Moreover, repeated exposure to antimicrobial agents during earlier treatments may induce or enhance secondary resistance, thereby reducing the efficacy of subsequent rescue regimens and increasing the likelihood of treatment failure. Although vonoprazan–minocycline/amoxicillin dual therapy is less affected by such resistance patterns, persistent infection might reflect multidrug-resistant *H. pylori* strains or poor adherence.

Notably, in this study, there was one case in both the VA group and the VM group where treatment failed with the respective regimen, and subsequent rescue treatments with the VM and VA regimens, respectively, were successful (Table 3). This suggested that there might be no cross-resistance between amoxicillin and minocycline, making them viable complementary options for treatment failure.

Dizziness emerged as the most common side effect in the VM group, and there were also some less common but potentially serious adverse effects, such as hypotension (one case) and low-grade fever (one case). This was consistent with previous reports on quadruple regimens containing minocycline [9], indicating that caution was warranted when using the minocycline-containing regimen [6,15]. Minocycline-induced dizziness was primarily related to higher serum concentrations of the drug, which were significantly higher in women than in men. This might be due to women’s smaller body size, leading to greater drug exposure. Gender and body size might be important risk factors for dizziness [20]. Previous study showed that a daily dose of 150 mg of minocycline (75 mg twice daily) had similar side effects to 200 mg (100 mg twice daily), with fewer cases of nausea at the lower dose. Based on this, a 14-day regimen of 100 mg daily was likely safe [21]. The results highlighted the higher incidence of TEAEs in the VM group compared to the VA group, underscoring the need for careful monitoring of adverse effects, particularly dizziness.

The success of *H. pylori* eradication depends heavily on the intensity, consistency, and duration of acid suppression. Although amoxicillin-based dual therapies were proposed decades ago [22], their eradication rates only became stable and improved when paired with double-dose PPIs or standard doses of P-CABs, leading to their more widespread clinical adoption in recent years [3]. Potent acid suppression broadens the scope of dual therapy, enabling a single sensitive antibiotic to achieve successful eradication when gastric pH is elevated to 6 or above through the use of P-CABs [23]. This may explain the differing outcomes of vonoprazan–tetracycline (VT) dual therapy compared to omeprazole–tetracycline dual therapy in earlier studies [5,24,25]. It had facilitated a treatment approach combining “potent acid suppression + a single sensitive antibiotic,” bringing *H. pylori* therapy closer to the principles applied in managing other infectious diseases. This approach could represent a potential strategy for the future management of *H. pylori* infections.

Currently, amoxicillin-based dual therapy is being increasingly adopted in China due to its effectiveness. However, this widespread use might lead to a rise in amoxicillin-resistance in the coming years. Dual therapies incorporating antibiotics with lower resistance rates, such as tetracycline or minocycline, could offer viable alternatives to address this situation. These regimens might also provide effective rescue options for patients who fail amoxicillin-based dual therapies.

## 5. Limitations

This RCT was designed based on our previous research which validated the efficacy of VT dual therapy. Minocycline provides a key advantage over tetracycline due to its broader availability. Additionally, its twice-daily dosing schedule offers greater convenience for patients compared to the three-times-daily regimen required for tetracycline. Our results indicated that VM dual therapy could be a viable treatment option. However, several limitations must be acknowledged. First, this study was conducted at a single center and used an open-label design with a limited sample size, which hinders the ability to make definitive conclusions about its efficacy and safety. Larger, multi-center studies are necessary to confirm these findings. Second, the comparison was confined to amoxicillin-based dual therapy, which provided an initial assessment of efficacy and safety but did not explore the potential of VM dual therapy as an alternative to tetracycline-based regimens. A direct comparison with tetracycline-based dual therapies, such as the vonoprazan-tetracycline (VT) regimen, would yield more clinically relevant insights. Third, the study did not include data on *H. pylori* isolation or antimicrobial susceptibility, which limits the interpretation of the findings. Finally, although dual therapies may have varying effectiveness across different populations, this study did not investigate such variations, underscoring the need for further research in diverse demographic and regional contexts [26,27].

## 6. Conclusions

Vonoprazan and minocycline dual therapy (VM dual therapy), comprising vonoprazan 20 mg twice daily and minocycline 100 mg twice daily for 14 days, proved to be both effective and safe as a first-line and rescue treatment for *H. pylori* infection, demonstrating comparable eradication rates to amoxicillin-based dual therapy for *H. pylori* treatment, but with a relatively higher incidence of adverse events. It might be more suitable for patients who are not candidates for amoxicillin-based regimens due to allergy or resistance. Further large-scale trials are needed to better evaluate the overall safety and efficacy profile of minocycline dual therapy. Combining potent acid suppression with well-tolerated and effective antibiotics could offer a promising direction for future research in the treatment of *H. pylori* infection. Larger-scale studies are warranted to confirm its efficacy, with safety remaining a critical focus in future evaluations. Dual therapy combining strong acid suppression and sensitive antibiotics might serve as a promising direction for future research in *H. pylori* treatment.

## Figures and Tables

**Table 1:** Baseline characteristics and demographics.

Characteristics	First-Line Treatment (*n* = 60)		Rescue Treatment (*n* = 60)	
	VA Group (*n* = 30)	VM Group (*n* = 30)	VA Group (*n* = 30)	VM Group (*n* = 30)
Age, y, mean (SD)	52.3 (4.2)	47.1 (9.9)	52.3 (6.4)	47.9 (17.0)
Sex (M/F)	15/15	15/15	16/14	10/20
Body weight (mean, SD) kg	65.5 (3.5)	65.5 (2.1)	64.7 (14.1)	62.9 (8.5)
BMI (mean, SD) kg/m^2^	23.8 (0.5)	23.8 (2.0)	23.2 (3.6)	22.9 (1.2)
Cigarette smoking	4 (13.3%)	1 (3.3%)	2 (6.6%)	1 (3.3%)
Alcohol drinking	6 (20.0%)	1 (3.3%)	4 (13.3%)	2 (6.6%)
Family history of gastric cancer [11]	5 (16.7%)	1 (3.3%)	2 (6.6%)	6 (20.0%)
Endoscopy diagnosis				
Gastritis	21 (70.0%)	25 (83.3%)	25 (83.3%)	25 (83.3%)
Peptic ulcers	7 (23.4%)	5 (16.7%)	5 (16.7%)	4 (13.3%)
Gastric cancer	1 (3.3%)	0	0	1 (3.3%)
MALToma	1 (3.3%)	0	0	0
Times of treatment failure (mean)	-	-	1.93	2
Loss of follow-up	0	0	1 (3.3%)	0
Quit treatment	0	2 (6.7%)	0	1 (3.3%)
Adherence, *n*/N (%)	30/30 (100%)	28/30 (93.3%)	29/30 (96.7%)	29/30 (96.7%)

**Table 2:** Eradication rate of each group.

Analysis	Total (*n* = 120)			First-Line Treatment (*n* = 60)			Rescue Treatment (*n* = 60)		
	VA Group (*n* = 60)	VM Group (*n* = 60)	*p* Value	VA Group (*n* = 30)	VM Group (*n* = 30)	*p* Value	VA Group (*n* = 30)	VM Group (*n* = 30)	*p* Value
ITT	91.7% (55/60)	83.3% (50/60)	0.17	96.7% (29/30)	90.0% (27/30)	0.30	86.7% (26/30)	76.7% (23/30)	0.32
95% CI	81.9–96.4%	72.0–90.7%		83.3–99.4%	74.4–96.6%		70.3–94.7%	59.1–88.2%	
PP	93.2% (55/59)	87.7% (50/57)	0.31	96.7% (29/30)	96.4% (27/28)	0.96	89.7% (26/29)	79.3% (23/29)	0.28
95% CI	83.8–97.3%	76.8–93.9%		83.3–99.4%	82.3–99.4%		73.6–96.4%	61.6–90.2%	

**Table 3:** Details of patients who failed in treatment.

Group	Subgroup	No.	Sex	Age (y)	Body Wight (kg)	BMI kg/m^2^	Adverse Events	Medication Days	Times of Previous Failure	DOB Before Treatment	DOB After Treatment	Rescue Treatment After Failed in Trial
VA group (*n* = 4)	First-line treatment (*n* = 1)	18	M	34	95	31.74	No	14	0	59.1	19.8	NA
	Rescue treatment (*n* = 3)	1	F	49	55	20.20	No	14	3	45.3	55.7	NA
		3	F	46	49	19.14	No	14	2	18.8	8.9	NA
		14	F	47	54	19.83	No	14	1	36.7	26.8	Successful in VM dual therapy
VM group (*n* = 10)	First-line treatment (*n* = 3)	1 (quit)	F	45	59	22.21	Dizziness + nausea + fatigue + Decreased blood pressure + Decreased heart rate	4	0	18	12.5	Successful in VA dual therapy
		3 (quit)	F	47	84	31.23	Dizziness + nausea + vomiting + tenderness of breasts	4	0	18	NA	NA
		30	M	31	56	19.38	No	14	0	42.6	6.2	NA
	Rescue treatment (*n* = 7)	2	F	38	57	22.31	No	14	2	17.8	15.6	NA
		5	F	36	57	20.20	No	14	2	52.4	35.4	NA
		11 (quit)	F	28	47	24.34	Low fever + rash	11	1	38.0	NA	NA
		12	F	38	55	22.31	No	14	2	53.4	47.1	NA
		20	F	57	59	19.27	No	14	1	10.2	7.4	NA
		21	M	27	72	24.91	No	14	2	14.5	19.3	NA
		25	M	61	60	22.04	No	14	4	38.9	15.3	NA

**Table 4:** Overview of adverse events (AEs).

Variables	VA Group (*n* = 60)	VM group (*n* = 60)	*p* Value
Total, *n*/N (%)	6/60 (10.0%)	18/60 (30.0%)	0.006
AEs variety			
Dizziness	0	11 (18.3%)	
Nausea	0	4 (6.7%)	
Abdominal discomfort	2 (3.3%)	3 (5.0%)	
Rash	1 (1.7%)	1 (1.7%)	
Constipation	1 (1.7%)	1 (1.7%)	
Diarrhea	1 (1.7%)	0	
Tenderness of breasts	0	1 (1.7%)	
Low fever	0	1 (1.7%)	
Knee joint redness and swelling	1 (1.7%)	0	
Vomiting	0	1 (1.7%)	
Abdominal pain	0	1 (1.7%)	
Fatigue	0	1 (1.7%)	
Decreased blood pressure+ Decreased heart rate	0	1 (1.7%)	
Anxiety	0	1 (1.7%)	
Manifestation of Cases who discontinued due to AEs (*n* = 3)			
Dizziness +nausea + fatigue + Decreased blood pressure + Decreased heart rate	0	1	
Dizziness + nausea + vomiting + tenderness of breasts	0	1	
Low fever + rash	0	1	
Discontinued due to AEs	0	3/60 (5.0%)	0.08
Adherence, *n*/N (%)	59/60 (98.3%)	57/60 (95.0%)	0.31

## Data Availability

The data that support the findings of this study are not publicly available due to privacy and ethical restrictions. However, they can be obtained from the corresponding author upon reasonable request. Data sharing is subject to the approval of the institutional ethics committee and may require a data use agreement.

## Abbreviations

The following abbreviations are used in this manuscript:

VA (dual) group: dual therapy group including vonoprazan 20 mg b.i.d. + amoxicillin 1.0 g t.i.d. for 14 days; VM (dual) group: dual therapy group including vonoprazan 20 mg b.i.d. + minocycline 500 mg b.i.d. for 14 days; VT (dual) therapy: vonoprazan-tetracycline dual therapy; Data are *n* (%), or mean (SD, standard deviation); BMI: body mass index; AEs: adverse events.
